# Mind the Gap

**DOI:** 10.1192/bjo.2026.11519

**Published:** 2026-06-30

**Authors:** Joshua Withers, Sana Fatima, Sherlie Arulanandam, Ulrich Muller-Sedgwick

**Affiliations:** 1 Avon & Wiltshire Mental Health Partnership NHS Trust, Swindon, United Kingdom; 2 Bradford District Care NHS Foundation Trust, Bradford, United Kingdom; 3 Government of Jersey, St Helier, United Kingdom

## Abstract

**Aims:**

Evaluate current practice in diagnosis and management of neuro developmental disorders (NDD) including Autism (ASD) and Attention-Deficit-Hyperactivity Disorder (ADHD) in Early Intervention Psychosis (EIP) services across the UK given frequent comorbidity of ADHD/ASD and Psychosis.

Gather data on care provision, use of third sector services and ascertain training and confidence in NDD within EIP services.

Highlight the need for robust NDD training to facilitate diagnosis and management within mental health teams, reducing pressure on services.

**Methods:**

An online survey was disseminated to members in EIP services through RCPsych General Adult faculty and the EIP network. Data collected included prefilled and free text responses regarding EIP team demographics, current NDD assessment practice, care provided (including medication), use of third sector services, level of NDD training and, confidence assessed on a Likert scale. Quantitative and Qualitative data was analysed to capture key themes.

**Results:**

55 responses from 36 trusts across England and Wales were received; 85% (n=46) were EIP teams, 60% serving mixed urban/rural populations.

ADHD: Assessment: 75% (n=41) referred externally for assessment, with only 9% (n=5) completing assessments within EIP teams. A further 13%(n=7) shared assessments with another trust team.

Pharmacology: 47% (n=26) relied on third sector prescribers to prescribe ADHD medication, 15% (n=8) prescribed within teams, whilst 30% (n=17) used shared care agreements or prescribed together with another trust team.

ASD: Assessment: 70% (n=39) of assessments were completed by external services; only 7% (n=4) were conducted within EIP teams.

Post-diagnostic support: 45% (n=25) only provided signposting advice and 19% (n=10) offered no support.

NDD Training: 49% (n=27) reported no formal NDD training, of the 40% (n=22) that did, qualifications included ADOS, ADI-R and DIVA. Key barriers were lack of NDD skills and training opportunities, alongside funding and commissioning constraints.

Confidence: Confidence in NDD assessment and management was moderate, average confidence was 3/5 (42%) with remaining 29% spread both ≤2 or ≥4.

Qualitative Responses: Frequent themes included limited training and diagnostic inability. Overwhelming number of EIP teams rely on external services for NDD assessment. Teams with NDD training found it beneficial to services overall; others highlighted necessity for enhanced NDD diagnostic skills.

**Conclusion:**

The elevated risk of multiple adverse outcomes in this population highlights the critical importance of allocating resources for clinician training and upskilling in the diagnosis and management of NDD, equitable access to services, and effective screening, diagnosis, and treatment within existing EIP services.

